# Development of the Telehealth Usability Questionnaire (TUQ)

**DOI:** 10.5195/ijt.2016.6196

**Published:** 2016-07-01

**Authors:** BAMBANG PARMANTO, ALLEN NELSON LEWIS, KRISTIN M. GRAHAM, MARNIE H. BERTOLET

**Affiliations:** 1 DEPARTMENT OF HEALTH INFORMATION MANAGEMENT, SCHOOL OF HEALTH AND REHABILITATION SCIENCES, UNIVERSITY OF PITTSBURGH, PITTSBURGH, PENNSYLVANIA, USA; 2 COLLEGE OF HEALTH RELATED PROFESSIONS, STATE UNIVERSITY OF NEW YORK DOWNSTATE MEDICAL CENTER, BROOKLYN, NEW YORK, USA; 3 DEPARTMENT OF REHABILITATION SCIENCE AND TECHNOLOGY, SCHOOL OF HEALTH AND REHABILITATION SCIENCES, UNIVERSITY OF PITTSBURGH, PITTSBURGH, PENNSYLVANIA, USA; 4 DEPARTMENT OF EPIDEMIOLOGY, GRADUATE SCHOOL OF PUBLIC HEALTH, UNIVERSITY OF PITTSBURGH, PITTSBURGH, PENNSYLVANIA, USA

**Keywords:** Telehealth, Telehealth Usability Questionnaire (TUQ), Usability

## Abstract

Current telehealth usability questionnaires are designed primarily for older technologies, where telehealth interaction is conducted over dedicated videoconferencing applications. However, telehealth services are increasingly conducted over computer-based systems that rely on commercial software and a user supplied computer interface. Therefore, a usability questionnaire that addresses the changes in telehealth service delivery and technology is needed. The Telehealth Usability Questionnaire (TUQ) was developed to evaluate the usability of telehealth implementation and services. This paper addresses: (1) the need for a new measure of telehealth usability, (2) the development of the TUQ, (3) intended uses for the TUQ, and (4) the reliability of the TUQ. Analyses indicate that the TUQ is a solid, robust, and versatile measure that can be used to measure the quality of the computer-based user interface and the quality of the telehealth interaction and services.

In order to reap the potential benefits of telehealth technologies, the delivery system has to be usable for both patients and clinicians. Usability is the extent to which a product can be used by specified users to achieve specified goals with effectiveness, efficiency, and satisfaction in a specified context of use (ISO, 1992). This construct is a key to making systems easy to learn and easy to use (Nielsen & Mack, 1994). Measuring the usability of telehealth technology offers a way to evaluate and improve the effectiveness of both the technology and services delivered.

Traditionally, telehealth and telemedicine have been conducted over videoconferencing technologies such as Cisco-Tandberg and Polycom. Systems such as Cisco-Tanberg and Polycom are designed for the sole purpose of videoconferencing. However, recent advances in technologies allow videoconferencing to be conducted using multi-purpose computer technologies. Examples include VSee, Adobe Connect, and Cisco WebEx. These multipurpose technologies allow consumers to deliver telehealth using computer software applications instead of traditional videoconferencing systems which are primarily hardware based. In addition to multi-purpose technology, there have been software applications developed specifically for telehealth purposes. These include the Versatile Integrated System for Telerehabilitation (VISYTER) (Parmanto et al., 2010) and EHAB (Peel, Russel, & Gray, 2011) systems.

A review of the literature found a number of telehealth questionnaires developed mostly in the early 2000s, including the Telemedicine Satisfaction Questionnaire (TSQ) (Yip, Chang, Chan, & MacKenzie., 2003), Telemedicine Patient Questionnaire (TMPQ) (Demeris et al., 2000, 2004), and Telemedicine Satisfaction and Usefulness Questionnaire (TSUQ) (Bakken, 2009). However, the questionnaires developed in these previous studies were designed to evaluate special purpose videoconferencing technologies. In order to reap the potential benefits of current telehealth technologies, the delivery system has to be usable for both patients and clinicians. The questionnaires commonly employed to measure usability of a telehealth system, including user experience and satisfaction with various aspects of the technology or service, typically use a Likert scale approach to assessment (Mair & Whitten, 2000). Since telehealth technology connects clinicians and patients over a distance, the usability questionnaire is designed to measure the quality of the interactions between two sites (e.g., audiovisual quality, quality of communications, ease of use of the equipment) (Houston & Burton, 1997), and the overall impression of the service (i.e., level of comfort and satisfaction with the telemedicine encounter) (Bakken et al., 2006; Yip et al., 2003).

The objective of the present study is to report on the development and reliability assessment of a new usability tool, the Telehealth Usability Questionnaire (TUQ). The TUQ combines items from existing telehealth questionnaires with those from computer usability questionnaires, and was designed to be a comprehensive questionnaire that covers all usability factors (i.e., usefulness, ease of use, effectiveness, reliability, and satisfaction). The TUQ is intended for both clinicians and patients. In addition, the TUQ is intended for use with various types of telehealth systems, including the traditional videoconferencing systems, computer-based systems, and the new generation of mobile telehealth systems. As such, the TUQ utilizes questions that can be modified to correctly address the participants (clinicians or patients) and the telehealth system.

## METHODS

### QUESTIONNAIRE DEVELOPMENT

Development of the TUQ consisted of four phases: (1) literature review; (2) construct development; (3) item development; and (4) examination of reliability. Each of these phases will be covered in the subsequent sections.

### BACKGROUND ON EXISTING QUESTIONNAIRES

A literature review was conducted to identify existing questionnaires that have been widely used in the evaluation of telemedicine and computer/information technology. Identified questionnaires that were used as models for the TUQ were primarily from two fields: telemedicine and computer and information technology.

In the field of telemedicine the following questionnaires were identified: the Telemedicine Satisfaction Questionnaire (TSQ) (Yip et al., 2003), Telemedicine Patient Questionnaire (TMPQ) (Demeris et al., 2000, 2004), and Telemedicine Satisfaction and Usefulness Questionnaire (TSUQ) (Bakken, 2009). Telemedicine questionnaires focus on three factors of usability: usefulness, satisfaction, and interaction quality between patient and clinician over telemedicine technology. The TSQ clearly addresses the three usability factors central to telehealth. For example, it includes items unique to telemedicine such as audio and video quality. TSQ is a questionnaire designed specifically for telemedicine systems. TSQ was also designed for traditional interactive videoconferencing systems such as Polycom or Cisco Tandberg. One main difference between traditional videoconferencing systems and new generation computer-based systems is that the former type of system does not have a user interface that clinicians and patients interact with, which is the case with computer-based systems such as VSee. The traditional videoconferencing systems are usually setup by a technician, and the user (patient and clinician) does not need to know how to setup and interact with the system. This means that the TSQ lacks the items related to interface quality that are important for computer/software-based telehealth. However, because items of the TSQ so clearly address the usability factors central to telehealth, it was identified as a primary source of questionnaire items for the TUQ.

In the field of information and computer technology the following questionnaires were identified: the Technology Acceptance Model (TAM) (Davis 1993), and the IBM Post-Study System Usability Questionnaire (PSSUQ) developed by Lewis (1995). The TAM (Davis 1993) describes the relationships between perceived qualities of system usage, affective attitude, and behavioral responses to the system. This questionnaire is used widely in the business information arena. We derived questions related to the usability factors of usefulness and ease of use from the TAM. The PSSUQ measures system usability via a multitude of aspects, including system function, information and interface quality, to users’ satisfaction level. The evaluation covers the standards of effectiveness, efficacy and satisfaction (Lewis, 1995). From the PSSUQ, we derived items for ease of use, interface quality, reliability, and satisfaction.

### USABILITY ATTRIBUTES OF A TELEHEALTH SYSTEM

The TUQ was designed to be a comprehensive questionnaire that covers all usability factors, including usefulness, ease of use, effectiveness, reliability, and satisfaction. The following is a brief description of each of the usability factors assessed in the TUQ.

#### USEFULNESS

Usefulness refers to the users’ perception of how the telehealth system functions to provide a healthcare interaction/service similar to the traditional in-person encounter. The system is useful when it works and has positive effects on clinical outcomes or reduces clinical cost (Ekeland, Bowes, & Flottorp, 2010).

#### EASE OF USE AND LEARNABILITY

The system should be easy to learn and use to facilitate rapid work completion (Chen et al., 2009). A system that is easy to learn allows users to build on their knowledge without deliberate effort. For example, a system with intuitive icons is easier to use and to learn than command-based system.

#### INTERFACE QUALITY

Interface quality measures the interaction between the patient and the telemedicine technology or computer system. This includes the quality of the graphical user interface, the ease of navigation, and an overall impression of how the patient interacts with the telehealth system. This usability attribute was not part of previous telemedicine questionnaires because the telemedicine systems in the past did not have an interface; they were primarily just hardware that needed to be turned on. The Interface Quality sub-scale deals with how pleasant the system was to use for the consumer. It measures if s/he liked the system and if the system had all the functionality and capabilities s/he expected. For example, systems like VSee have a graphical user interface, while most old videoconferencing systems primarily consist of hardware.

#### INTERACTION QUALITY

Interaction quality measures patient interactions with the clinician, including the quality of the audio and the video, and the similarity of the telehealth interaction between patient and clinician to an in-person interaction. This construct is unique to telehealth and has been the focus of many of the telemedicine questionnaires (Demeris, 2000; Yip et al., 2003).

#### RELIABILITY

Reliability refers to how easily the user can recover from an error and how the system provides guidance to the user in the event of error. For example, if a user clicks a wrong button, the system provides a means to undo the error or to back track. Ideally, telehealth systems should be as reliable as in-person service. Reliability and validity of data transmission are essential to the safety of patients (Schlachta-Fairchild, Elfrink, & Deickman, 2008).

#### SATISFACTION AND FUTURE USE

This factor is related to overall satisfaction of the user with the telehealth system and how willing the user would be to use the system in the future.

Table 1 shows the usability components of the TUQ and questionnaire items for each. The table also shows the source of the questionnaire items. The telehealth-related questionnaires are primarily taken from TSQ (Yip et al., 2003). These items are related to usefulness, interaction quality, and satisfaction and future use. The items related to computer and user interface are primarily taken from PSSUQ (Lewis, 1995); these items elicit information about ease of use and learnability, interface quality, satisfaction and future use. The items in the reliability component that are taken from TSQ are related to the reliability of telehealth service, and those from PSSUQ are related to system reliability. The satisfaction and future use component includes questions from both TSQ and PSSUQ. Questionnaire items on ease of use and learnability were also borrowed from TAM, and items on usefulness were also taken from TAM and use similar wording.

## USABILITY FACTORS

The TUQ uses a broader definition of usability that takes into account the utility and the usability of the technology. Utility refers to whether the technology’s functionality does what users need (Nielsen, 2012). Usability is the extent to which a product can be used by users to achieve specified goals with effectiveness, efficiency and satisfaction (ISO, 1992). Early work in telehealth usability evaluation was primarily focused on patient satisfaction (Aoki et al., 2003; Heinzelmann, Williams, Lugn, & Kvedar, 2005), while later work incorporated satisfaction, usefulness, ease of use, and interaction quality (Bakken, 2006; Demiris, 2000, 2001, 2004; Yip et al., 2003), all of which are measures of effectiveness. The TUQ usability factors include usefulness, ease of use, effectiveness, reliability, and satisfaction. The relationship among the TUQ usability components, questionnaire items, and usability factors is depicted in Table 2.

### CONTENT VALIDITY

Because the questionnaire items included in the TUQ were combined from existing sources in telemedicine and computer software interface, the content validity of the questionnaire items was reported in previous studies (Bakken, 2006; Lewis, 1994; Yip et al., 2003). Additionally, the content validity of the TUQ has been shown in previous studies (Parmanto et al., 2011; Schutte et al., 2013; Yu et al., 2015a, 2015b).

### CONTENT RELIABILITY

#### PARTICIPANTS

Fifty-three participants (21 males and 32 females) took part in this study. Participants were recruited from the University of Pittsburgh and included individuals with (56.6%) and without (43.4%) experience utilizing telehealth technology. To be included in the study participants could not have completed the TUQ within the last three months. Table 3 provides characteristics of the participants.

## PROCEDURE

Basic demographic information was collected via interview, and the TUQ was completed independently by participants. All participants were directed to complete the TUQ based on their experience with the VISYTER system. Two participant perspectives were needed (clinicians and clients). Participants who regularly used telehealth technology and identified as “clinicians” were asked to complete the TUQ based on their most recent interaction with the VISYTER application. Participants who had never or did not regularly use telehealth technology were asked to take part in a simulated telehealth session as a client. The simulated telehealth session was designed to approximate an initial session that would occur between a rehabilitation practitioner and a new client. This allowed simulation participants to complete the TUQ from the “client” perspective, as they assumed the client role. These participants interacted with study staff, who assumed the clinician role via VISYTER. After completing the simulated telehealth session, both sets of participants (clinicians based on their prior experience and clients based on their experiences with the simulation) were asked to complete the TUQ.

## DATA ANALYSIS

The TUQ ratings by factor were compared using Cronbach’s coefficient alpha. This statistic is most often used as a measure of internal consistency and is used to evaluate if items in a scale are measuring the same construct (Portney & Watkins, 2009). Guidelines for evaluating Cronbach’s coefficient alpha are presented in Table 4.

## RESULTS

All usability attributes of the TUQ were found to have “good” to “excellent” reliability. Raw and standardized Cronbach’s coefficient alpha values for each are presented in Table 5. Refer back to Table 2 to view the subscales of the TUQ.

## DISCUSSION

The TUQ was developed in response to the ever-evolving technology within telehealth today. There is a need for a usability measure that exhibits the attributes of usefulness, ease of use and learnability, interface quality, interaction quality, reliability, satisfaction, and future use. These salient features must be present in measuring computer-based telehealth technologies. Building on the best measures currently available in telehealth and in information technology and computer science, we have designed the TUQ to include the above-mentioned attributes and to be psychometrically robust.

The TUQ has strong content validity because it incorporates items from the best current measures in telehealth, which have withstood the scrutiny of rigorous prior validation studies, and it has been used in previous studies conducted by several of these authors. The TUQ’s reliability, specifically internal consistency, is more than adequate. Its internal consistency was evaluated directly as a part of this investigation. The first step in the reliability examination was to develop a protocol to vary the TUQ’s standard language when administered orally to a targeted respondent group to ensure optimal understanding. Secondly, a “systems check” was performed by administering the TUQ using various telecommunication systems to ensure its operational integrity and quality when used in various technology contexts. Finally, we tested the TUQ’s internal consistency using Cronbach’s coefficient alpha. It performed remarkably well, with alpha values that substantially exceeded the acceptable range in all domains: usefulness, ease of use, effectiveness, reliability, and satisfaction. Additionally, the associated eigenvalues, factor loadings, and percentages of variation explained by each domain were strong.

Our development efforts and the analyses implicit in those efforts have revealed that the TUQ is a solid, robust, and versatile measure. It is based on the best usability questionnaires available, able to respond to the latest technology changes in telehealth, incorporates both client and clinician interface needs in the delivery of clinical services via telehealth, and addresses all of the relevant dimensions of usability. Its prognosis is strong; more widespread use in telehealth studies going forward will be the ultimate test of its effectiveness. Given the increase in pervasiveness of telehealth in the delivery of clinical services from a distance, along with the rise in use of computer-based systems that rely on software and a computer interface as the model of delivering telehealth, the TUQ will be valuable for measuring usability.

## Figures and Tables

**Table 1:** Usability Components and Questionnaire Items and Their Source

Components	Questionnaire Items	TAM	TSQ	PSSUQ
Usefulness				
1	Telehealth improves my access to healthcare services	S	Y	
2	Telehealth saves me time traveling to a hospital or specialist clinic	S	Y	
3	Telehealth provides for my healthcare needs	S	Y	
Ease of Use and Learnability				
1	It was simple to use this system	Y	S	Y
2	It was easy to learn to use the system	Y		Y
3	I believe I could become productive quickly using this system	Y		Y
Interface Quality				
1	The way I interact with this system is pleasant			Y
2	I like using the system			Y
3	The system is simple and easy to understand	S		Y
4	This system is able to do everything I would want it to be able to do	S		Y
Interaction Quality				
1	I could easily talk to the clinician using the telehealth system		Y	
2	I could hear the clinician clearly using the telehealth system		Y	
3	I felt I was able to express myself effectively		Y	
4	Using the telehealth system, I could see the clinician as well as if we met in person		Y	
Reliability				
1	I think the visits provided over the telehealth system are the same as in-person visits		Y	
2	Whenever I made a mistake using the system, I could recover easily and quickly	S		Y
3	The system gave error messages that clearly told me how to fix problems			Y
Satisfaction and Future Use				
1	I feel comfortable communicating with the clinician using the telehealth system		Y	Y
2	Telehealth is an acceptable way to receive healthcare services	S	Y	Y
3	I would use telehealth services again		Y	
4	Overall, I am satisfied with this telehealth system		Y	Y

Note. Y = taken from the questionnaire with no or slight change; S = Similar item with different wording exists in the questionnaire.

**Table 2:** Usability Components, Questionnaire Items, and Usability Factors

Components	Factors	Usefulness	Ease of use	Effectiveness	Reliability	Satisfaction
**Usefulness**						
1	Telehealth improves my access to healthcare services	X				
2	Telehealth saves me time traveling to a hospital or specialist clinic	X				
3	Telehealth provides for my healthcare needs	X				
**Ease of Use & Learnability**						
1	It was simple to use this system		X			
2	It was easy to learn to use the system		X			
3	I believe I could become productive quickly using this system		X			
**Interface Quality**						
1	The way I interact with this system is pleasant		X			
2	I like using the system		X			
3	The system is simple and easy to understand		X			
4	This system is able to do everything I would want it to be able to do			X		
**Interaction Quality**						
1	I could easily talk to the clinician using the telehealth system			X		
2	I could hear the clinician clearly using the telehealth system			X		
3	I felt I was able to express myself effectively			X		
4	Using the telehealth system, I can see the clinician as well as if we met in person			X		
**Reliability**						
1	I think the visits provided over the telehealth system are the same as in-person visits				X	
2	Whenever I made a mistake using the system, I could recover easily and quickly				X	
3	The system gave error messages that clearly told me how to fix problems				X	
**Satisfaction and Future Use**						
1	I feel comfortable communicating with the clinician using the telehealth system					X
2	Telehealth is an acceptable way to receive healthcare services					X
3	I would use telehealth services again					X
4	Overall, I am satisfied with this telehealth system					X

**Table 3:** Participant Characteristics

Characteristic	n	Percent (%)
Gender		
Male	21	39.6
Female	32	60.4
Highest Level of Education		
Completed some college	17	32.1
Associate’s degree	1	1.9
Bachelor’s degree	6	11.3
Completed some postgraduate	11	20.8
Master’s degree	11	20.8
PhD, law or medical degree	7	13.2
Race/Ethnicity		
African American	3	5.6
Asian	5	9.5
Hispanic	2	3.7
Caucasian	42	79.2
Other	1	2
Experience with Telehealth		
None	23	43.4
Less than 3 months	8	15.1
3 to 6 months	5	9.5
6 months to 1 year	2	3.7
More than 1 year	15	28.3

**Table 4:** Cronbach’s Coefficient Alpha Ranges of Acceptability

Scale	Rating
α ≥ 0.9	Excellent
0.8 ≤ α < 0.9	Good
0.7 ≤ α < 0.8	Acceptable
0.6 ≤ α < 0.7	Questionable
0.5 ≤ α < 0.6	Poor
α < 0.5	Unacceptable

**Table 5:** Internal Consistency of the TUQ Subscales

Variable	Cronbach Coefficient Alpha	
	*Raw*	*Standardized*
Usefulness	0.83	0.85
Ease of Use	0.92	0.93
Effectiveness	0.86	0.87
Reliability	0.79	0.81
Satisfaction	0.91	0.92
